# Increased Intra-Individual Variability as a Marker of Executive Dysfunction in Generalized Anxiety Disorder

**DOI:** 10.3389/fpsyt.2022.532778

**Published:** 2022-03-22

**Authors:** Yongju Yu, Haiyan Xu, Yuanyuan Xu, Fang Lu, Min Li

**Affiliations:** ^1^Department of Social Work, School of International Law and Sociology, Sichuan International Studies University, Chongqing, China; ^2^Department of Military Psychology, School of Medical Psychology, Army Medical University, Chongqing, China; ^3^School of Nursing, Army Medical University, Chongqing, China

**Keywords:** intra-individual variability (IIV), generalized anxiety disorder (GAD), trait anxiety, raw intra-individual standard deviation (SD), reaction time coefficient of variation (RTCV), mean absolute deviation (MAD)

## Abstract

Recent studies suggest that individual difference in intra-individual variability (IIV) of reaction times is an important indicator of attentional executive control. However, there are few existing studies on the executive control of high trait-anxious individuals assessed by using reaction time variability. This study assessed whether executive functions are impaired among clinical and non-clinical trait-anxious individuals indicated by IIV. The cross-reliability and discriminative power of three IIV parameters (raw intra-individual standard deviation, SD; reaction time coefficient of variation, RTCV; and mean absolute deviation, MAD) were compared. Twenty-five non-clinical individuals with low trait anxiety (LTA), 31 non-clinical individuals with high trait anxiety (HTA), and 19 clinical patients diagnosed with generalized anxiety disorder (GAD) finished self-reported measures, an emotional spatial-cuing task, and a non-emotional arrow flanker task. In the emotional task, GAD patients had significantly slower response speed, lower accuracy, and greater IIV parameters than the LTA and HTA groups. In the non-emotional task, the GAD group exhibited poorer processing efficiency, greater SD and RTCV, and intact performance effectiveness. RTCV is suggested to be a better marker of executive dysfunction than SD and MAD due to its good discriminative power and reliability as well as less affected by reaction times.

## Introdction

Intra-individual variability (IIV) of reaction times (RTs) refers to the short-term changes or fluctuations in an individual's performance during a task (1). Recent studies suggest that individual difference in IIV is an important indicator of attention and cognitive control (2). It is well-known that individual sensitivity is periodically strengthened and weakened, i.e., attentional fluctuations. Apart from being affected by fatigue and distracting stimuli, attentional fluctuations are more affected by intrinsic neuronal activity, which is considered to be the main factor contributing to IIV (3). As a psychological noise, IIV of RTs reflects individuals' ability of sustained attention. Larger IIV means that task manipulation is characterized by poorer response efficiency, more attention errors, inability to maintain attention to a given task for a long time, or more maladaptive behaviors (4–6). This instability in cognitive control can show up over time in the changes of RTs across trials (7).

It has been found that IIV is superior to mean RT, operational errors, omission errors, and success inhibition ratio (4, 8) in measuring executive control function. Researchers have examined the psychometric characteristics of IIV to test its stability and reliability. Ode et al. found that the correlation coefficient of IIV was 0.68 with an intervening time interval of at least 2 weeks (5). In the same year, Saville et al. found that IIV of RTs showed good test–retest and odd–even reliability (9). These studies provide strong evidence that IIV is a relatively stable trait. Furthermore, it is also found that IIV represents a unitary construct, and it exhibits good consistency across not only different cognitive tasks, but also different sensory modalities (10).

The study on IIV was first initiated among individuals with attention deficit hyperactivity disorder (ADHD). In a meta-analytic review based on 319 studies (11), researchers found that children and adults with ADHD exhibit increased IIV relative to non-clinical groups. Furthermore, IIV reflects a stable feature of ADHD and other clinical disorders, which is robust to systematic differences across a wide range of tasks. An age-related decrease in the efficiency of executive control can result in an increase in performance variability in task conditions requiring the recruitment of executive control processes (12). Increased trial-to-trial IIV was highlighted among different types of clinical samples, such as ADHD (4), depressive disorder (13), and post-traumatic stress disorder (PTSD) (14). Swick et al. found veterans diagnosed with PTSD exhibited greater response variability due to their deficits in the sustained attention and top-down cognitive control processes, which, in turn, strengthened the maintenance of PTSD symptoms (7). Individuals with head injuries also demonstrated greater variability than the healthy controls (15, 16).

At present, increased IIV of RTs is also found among non-clinical examples, such as individuals with greater decline in perceptual speed and ideational fluency (17), cognitive aging (12, 18), and poorer physical performance (19). IIV measures were revealed to be closely related to falls and gait in the elderly (20). A study on community-dwelling adults showed that older and more depressed adults exhibited greater IIV, but not for mean RT in the visual search task (21). Based on the perceptual speed study, Papenberg et al. insisted that increased IIV can predict cognitive impairment in the elderly (22). Five empirical studies conducted by Ode et al. among college students revealed that individuals with higher variability exhibited less effective cognitive control and less controlled behaviors and were more prone to negative emotional experiences and depressive symptoms (5). Moreover, IIV is considered to be a unique cognitive function indicator independent of accuracy, and it is closely related to children's externalizing problems (6).

Although the close relationship between IIV and cognitive impairments is widely known, it still remains unknown whether this association exists in high-anxious individuals. Increasing evidence lends support to the notion that anxiety is associated with performance impairments on numerous tasks. According to attentional control theory (23, 24), anxiety impairs the efficiency of executive functions with performance effectiveness (the quality of performance generally indexed by response accuracy) and poor processing efficiency, which can be inferred from differences in reaction time (23). Furthermore, compared with performance effectiveness, processing efficiency may be more susceptible to anxious symptoms (24). Individuals with high trait anxiety may use more top-down control resources to compensate for their reduced processing efficiency to achieve the same level of behavioral performance as non-clinical individuals. Accordingly, this study examined the characteristics of attention executive control indicated by IIV among individuals with different levels of trait anxiety based on clinical and non-clinical samples.

Currently, there are several measures of IIV, such as the raw intra-individual SD, RTCV, MAD, residualized intra-individual SDs (ISD) using a regression procedure, the μ parameter of the ex-Gaussian distribution, the mean reciprocal RT (RecipMRT), and the μ parameter of the LATER model. However, it still remains inconsistent concerning which measure of IIV is most stable and reliable. Among these parameters, raw SD represents the discrete degree of individual RT, which is easy to calculate and understand. RTCV adjusts the raw intra-individual SD by intra-individual mean RT (20), which avoids the influence of mean RT. MAD is the mean deviation from the mean RT, which can better reflect the actual deviation of individual performance. These three parameters measure the spread of observations around the mean, irrespective of the direction of the deviation (9). Lövdén et al. report that RTCV produced the most stable and robust solutions (17), and Saville et al. find that SD and MAD were superior to other measures (9). Therefore, in this study, we compared these measures (SD, MAD, and RTCV) to explore the most stable and reliable indicator.

Previous studies show that individuals reporting more anxious symptoms performed significantly worse in threat-related tasks than those who had less anxious symptoms (25), whereas high-anxious individuals usually exhibited a comparable level of performance as low-anxious individuals in the neutral stimuli tasks or low cognitive load (23, 26). Accordingly, to examine whether there are differences in the performance for individuals with different levels of trait anxiety, an emotional spatial-cuing task with threat-related stimuli and an arrow flanker task with neutral stimuli were conducted in our study. Another focus of this study is whether IIV can distinguish high-anxious individuals from high-anxious ones due to its relative independence of accuracy. We speculate that high-anxious individuals may exhibit more increased IIV measures than the controls in both the emotional and the non-emotional tasks. Taken together, the aims of the present study are, therefore, two-fold. First, the executive control functions indicated by IIV among individuals with different levels of trait anxiety based on clinical and non-clinical samples (patients diagnosed with GAD from hospitals, healthy persons with low and high trait anxiety) were assessed in emotional and non-emotional tasks. Second, the across-task reliabilities and independence from RTs of different IIV parameters (SD, MAD, and RTCV) were compared in cognitive tasks.

## Materials and Methods

### Participants and Procedures

This study was approved by the Ethics Committee of Army Medical University, Chongqing, China. Experiments were conducted in three groups: non-clinical persons with LTA (the LTA group), non-clinical persons with HTA (the HTA group), and high trait-anxious patients diagnosed with GAD (the GAD group). The LTA and HTA groups were recruited from the local community by advertisement. Patients with GAD were recruited from the outpatient clinic of Xinqiao Hospital and Daping Hospital in Chongqing, China.

The classification criteria of trait anxiety depended on the results of pre-experiments in 1,539 healthy persons (27). Non-clinical participants with lower trait anxiety scores (≤ 33) were assigned to the LTA group, and the ones with higher trait anxiety scores (≥40) were assigned to the HTA group. All participants met criteria as follows: (1) able to read and understand the questionnaire; (2) had normal or corrected normal vision; (3) no evidence of substance abuse or dependence in the past 3 months. Besides that, the LTA and HTA individuals had no mental and cognitive disorders or brain injury. Patients with GAD were first-episode outpatients diagnosed by two licensed clinical psychologists. Psychiatric diagnoses were confirmed by using the Chinese version of the Mini-International Neuropsychiatric Inventory (28, 29). All GAD individuals had not taken psychiatric drugs in the last 3 months and had no treatment of rTMS or electroconvulsive therapy in the past 6 months. Importantly, given past findings that changes in IIV are associated with depressive symptoms and the high comorbidity of GAD and depression, clinically depressed patients and individuals with history of depression were excluded in the current study. Our study recruited 25 LTA individuals, 31 HTA individuals, and 19 patients with GAD. All participants completed written informed consent after a detailed explanation of aims and procedures of this study. All participants in the current study are Han nationality. Non-clinical participants completed questionnaires in the laboratory or classrooms, and GAD patients completed questionnaires in the hospital. Then, we analyzed the data and selected qualified participants. Individuals who met the inclusion criteria were invited to enter the laboratory and completed experimental tasks on computers within 1 week after qualification screening.

### Materials and Tasks

#### Self-Report Measures

Age, gender, highest level of education (1 = “less than high school,” 2 = “completed high school,” 3 = “college or bachelor's degree,” 4 = “master's or doctoral degree”), and past history of disease were reported. The trait subscale of Spielberger's State-Trait Anxiety Inventory (STAI_T) (30, 31) was used to measure the level of trait anxiety. This subscale consists of 20 items that can indicate individuals' tendency to perceive stressful situations as dangerous or threatening. Internal consistency was Cronbach's alpha = 0.938 for STAI_T in the current study.

#### Emotional Cognitive Task

The emotional spatial-cuing paradigm (32, 33) was adopted in our cognitive tasks. The target stimuli were presented on the left or right side of a fixation point. In half of the trials, a cue precedes the target at the same location (“valid cues”), whereas in the other half of trials, the cue is presented on the opposite side from the target (“invalid cues”). Half of the clues are neutral pictures (for example, a marble sculpture) and the others are negative ones (for example, a snake). There are 12 neutral and 12 negative pictures chosen from the standardized native Chinese Affective Picture System (CAPS) (34). Scores of pleasure, arousal, and dominance for all pictures rated on a 1–9 rating scale (1 = “not at all” and 9 = “very high”) can be obtained from the CAPS. In this study, pleasure scores for all negative pictures were <2.5, and pleasure scores for neutral pictures were between 4.5 and 5.5. Meanwhile, the scores of arousal degree were matched between neutral and negative pictures. Neutral pictures had significantly higher levels of pleasure [*t*_(22)_ = 24.17, *p* < 0.001] and dominance [*t*_(22)_ = 11.09, *p* < 0.001] than negative pictures, and no significant difference was found in arousal between neutral and negative pictures [*t*_(22)_ = 0.21, *p* = 0.391]. The sequence of events of the trial in this study was set according to the previous literature (32, 33). At the beginning of each trial, a fixation cross was presented in the center of the display together with two peripheral boxes, one to the left side and one to the right side. After 500 ms, a negative or neural cue picture showed up 400 ms in the left or right peripheral box (50% probability). No response was needed to the cue picture. Subsequently, to reduce expectation effect, there was a gap of either 50 or 800 ms that appeared at the center in one of the two peripheral boxes. Participants were instructed to press the “f” key as soon as they spotted the target at the left side and the “j” key at the other side. Stimuli were presented in a pseudo-random order and remained on the screen until the individual responded. The interstimulus interval was 1,000 ms. In total, the experiment had 176 trials, including 16 practice trials and 160 experimental trials.

#### Non-emotional Cognitive Task

Another cognitive task in the present study was the non-emotional arrow flanker task (35). Stimuli were presented in white against a black background on a computer screen. In each trial, the target arrow (1.05 × 1.37°) was surrounded by two horizontally arranged arrows on right and left sides: < < < < <, > > < > >, > > > > >, or < < > < < (25% probability, respectively). Participants were instructed to respond to the central target arrow by pressing a spatially compatible key on the computer keyboard (“f” or “j”) with their left or right index finger, respectively. At the beginning of each trial, a fixation cross displayed on the center of the screen for 500 ms. After that, the fixation cross was replaced by the stimulus. The entire stimulus array remained on the screen until the individual responded. Participants were encouraged to respond to the stimuli as quickly and accurately as possible. A varying interstimulus interval was set between 800 and 1500 ms. In total, the experiment had 217 trials, including 25 practice and 192 experimental trials. All the trials were presented in a pseudo-random order. The emotional and non-emotional cognitive tasks were performed in a standard ABBA sequence among participants.

### Data Analysis

The data were statistically analyzed using SPSS software version 20. First, the accuracy was calculated under different experimental conditions. Then, trials with wrong responses were removed from the data. Mean and standard deviation of RTs (MRT, SD, respectively) were computed. Accordingly, MAD and RTCV were also obtained. Specifically, MAD is the mean value of absolute deviation from the mean across all trials. Log unit scores of RT (LSRTs) were also computed across all trials for each individual, and then RTCV was obtained by dividing standard deviation of LSRTs with their mean value (5). SD, MAD, and RTCV for all trials with correct responses in the emotional and non-emotional cognitive tasks were computed separately for each individual.

To compare executive control function among the LTA, HTA, and GAD groups, a series of univariate analyses were conducted with the group as an independent variable, mean RTs, accurate rates, and three IIV parameters served as dependent variables, respectively. To compare the across-task reliabilities and the independence from RTs of three IIV parameters, after controlling for sociodemographic variables, partial correlation analyses were performed among mean RT, accuracy, SD, MAD, and RTCV in the emotional and non-emotional tasks. The significance level was set at *p* < 0.05 in our study.

## Results

### Demographics and Self-Report Data

The ages of the 75 participants ranged from 18 to 45 years (mean = 24.17, SD = 6.25). Among them, 72% were men, and 28% were women. As listed in Table 1, the LTA, HTA, and GAD groups did not significantly differ in gender [*F*_(2, 72)_ = 1.254, *p* = 0.291, $\eta_{p}{}^{2}$ = 0.063], age [*F*_(2, 72)_ = 1.627, *p* = 0.204, $\eta_{p}{}^{2}$ = 0.033], or education [*F*_(2, 72)_ = 2.044, *p* = 0.137, $\eta_{p}{}^{2}$ = 0.023]. Scores of trait anxiety significantly differed across groups [*F*_(2, 72)_ = 21.437, *p* < 0.001, $\eta_{p}{}^{2}$ = 0.841]. Further analyses indicated that the GAD group reported significantly higher levels of trait anxiety than the LTA and HTA groups (*p*s < 0.001). In addition, the HTA group exhibited significantly higher levels of trait anxiety than the LTA group (*p* < 0.001).

**Table 1:** Demographic and STAI-T data for participants (mean ± SD).

		**LTA (25)**	**HTA (31)**	**GAD (19)**	***P***
	Less than high school	0	0	4 (21.05%)	0.137
Education	Completed high school	22 (88.00%)	22 (70.97%)	10 (52.63%)	
	Junior college or bachelor's degree	3 (12.00%)	5 (16.13%)	4 (21.05%)	
	Graduate	0	4 (12.90%)	1 (5.26%)	
	Female	6 (24.00%)	7 (22.58%)	8 (42.10%)	0.291
	Age	23.2 ± 4.37	23.61 ± 5.57	26.37 ± 8.75	0.204
	STAI-T	29.00 ± 3.24	46.77 ± 5.05	59.63 ± 7.86	<0.001

### Comparisons in Executive Control Function Among Three Study Groups

Controlling for age, gender, and education level, univariate analyses were conducted with group as the independent variable and mean RT, accuracy, MAD, RTCV, and SD as the dependent variables for the emotional and non-emotional tasks, respectively. Descriptions of mean RT, accuracy, MAD, RTCV, and SD in each cognitive task are presented in Table 2. In the emotional cognitive task, results show that there were significant differences among the three groups in RT [*F*_(2, 68)_ = 3.512, *p* =.035, $\eta_{p}{}^{2}$ = 0.094] and in correct rate [*F*_(2, 68)_ = 14.418, *p* < 0.001, $\eta_{p}{}^{2}$ = 0.295]. *Post hoc* analysis suggests that the GAD group had significantly slower response speed and lower accuracy than the LTA and HTA groups (*p*s < 0.001), and there was no difference between the other two groups (*p*s > 0.05). In the non-emotional cognitive task, a significant difference was found among these three groups in RT [*F*_(2, 68)_ = 4.454, *p* = 0.0145, $\eta_{p}{}^{2}$ = 0.116], but not in accuracy [*F*_(2, 68)_ = 0.445, *p* = 0.643, $\eta_{p}{}^{2}$ = 0.013]. *Post hoc* analysis also found that the GAD group had significantly slower response speed than the LTA and HTA groups (*ps* < 0.001).

**Table 2:** Comparison of group differences in main study variables.

**Variable**	**LTA (*n* = 25)**	**HTA (*n* = 31)**	**GAD (*n* = 19)**	***F***	***P***	$\eta_{p}{}^{2}$
RT_T1	372.18 ± 61.88	379.53 ± 65.48	476.14 ± 102.70	3.512	0.035	0.094
RT_T2	473.91 ± 44.97	472.54 ± 55.86	597.18 ± 101.75	4.454	0.015	0.116
Accuracy_T1	99.58 ± 0.69	99.72 ± 0.58	97.83 ± 1.32	14.418	<0.001	0.295
Accuracy_T2	99.19 ± 0.99	99.29 ± 0.72	99.21 ± 1.47	0.445	0.643	0.013
MAD_T1	50.45 ± 15.88	50.00 ± 15.84	78.19 ± 24.55	6.160	0.003	0.153
MAD_T2	63.58 ± 14.05	63.56 ± 19.86	187.87 ± 200.01	1.541	0.222	0.043
RTCV_T1	2.96 ± 0.57	2.83 ± 0.46	3.42 ± 0.54	6.109	0.004	0.152
RTCV_T2	2.72 ± 0.55	2.68 ± 0.47	3.28 ± 0.74	5.450	0.006	0.138
SD_T1	71.71 ± 21.52	67.10 ± 19.31	102.51 ± 27.87	6.575	0.002	0.162
SD_T2	84.10 ± 17.96	83.66 ± 24.37	165.02 ± 55.84	6.867	0.002	0.168

*RT_T1, mean reaction time for all trials in emotional cognitive task; RT_T2, mean reaction time for all trials in non-emotional cognitive task; Accuracy_T1, accuracy for all trials in emotional cognitive task; Accuracy_T2, accuracy for all trials in non-emotional cognitive task; MAD_T1, mean absolute deviation for all trials in emotional cognitive task; MAD_T2, mean absolute deviation for all trials in non-emotional cognitive task; RTCV_T1, reaction time coefficient of variation for all trials in emotional cognitive task; RTCV_T2, reaction time coefficient of variation for all trials in non-emotional cognitive task; SD_T1, standard deviation of reaction times for all trials in emotional cognitive task; SD_T2, standard deviation of reaction times for all trials in non-emotional cognitive task. Sociodemographic variables (age, gender, and education level) were included as covariates for data analysis*.

According to univariate analyses, significant differences among these three groups in MAD [*F*_(2, 68)_ = 6.160, *p* < 0.01, $\eta_{p}{}^{2}$ = 0.153], RTCV [*F*_(2, 68)_ = 6.109, *p* < 0.01, $\eta_{p}{}^{2}$ = 0.152], and SD [*F*_(2, 68)_ = 6.575, *p* < 0.01, $\eta_{p}{}^{2}$ = 0.162] were obtained after controlling for sociodemographic variables in the emotional task. Further multiple comparisons showed that patients with GAD had significantly greater IIV parameters (MAD, RTCV, and SD) than the LTA and HTA groups (*p*s < 0.05), whereas no difference was found between the LTA and HTA groups (*p*s > 0.05). Similar results were obtained in the non-emotional task except for MAD [*F*_(2, 68)_ = 1.541, *p* = 0.222, $\eta_{p}{}^{2}$ = 0.043].

### Comparisons in Across-Task Reliabilities Among MAD, RTCV, and SD

Controlling for age, gender, and education level, partial correlation analyses were performed among MAD, RTCV, and SD in the emotional and non-emotional cognitive tasks to examine across-task reliabilities of three IIV parameters. As listed in Table 3, SD had the best across-task reliability (*r* = 0.62, *p* < 0.01), followed by RTCV (*r* = 0.53, *p* < 0.01), and the worst is MAD (*r* = 0.39, *p* < 0.01). Partial correlation analyses were also conducted among MAD, RTCV, SD, and mean RT. It was found that RT had stronger correlations with SD (*r* = 0.84 for the emotional task, *r* = 0.88 for the non-emotional task) and MAD (*r* = 0.87 for the emotional task, *r* = 0.76 for the non-emotional task) than with RTCV (*r* = 0.51 for the emotional task, *r* = 0.64 for the non-emotional task), which revealed that RTCV was more independent from RT than MAD and SD. Besides that, controlling for sociodemographic variables, trait anxiety was significantly related to MAD, RTCV, and SD (*p*s < 0.05) both in the emotional and non-emotional tasks.

**Table 3:** Partial correlations among different study variables.

	**RT_T1**	**RT_T2**	**ACC_T1**	**ACC_T2**	**MAD_T1**	**MAD_T2**	**RTCV_T1**	**RTCV_T2**	**SD_T1**	**SD_T2**
RT_T1	1									
RT_T2	0.71^**^	1								
ACC_T1	−0.48^**^	−0.57^**^	1							
ACC_T2	0.25^*^	0.08	0.16	1						
MAD_T1	0.87^**^	0.64^**^	−0.61^**^	0.08	1					
MAD_T2	0.45^**^	0.76^**^	−0.66^**^	−0.01	0.39^**^	1				
RTCV_T1	0.51^**^	0.41^**^	−0.52^**^	−0.12	0.84^**^	0.55^**^	1			
RTCV_T2	0.37^**^	0.64^**^	−0.47^**^	−0.17	0.47^**^	0.58^**^	0.53^**^	1		
SD_T1	0.84^**^	0.64^**^	−0.59^**^	0.06	0.98^**^	0.41^**^	0.88^**^	0.53^**^	1	
SD_T2	0.58^**^	0.88^**^	−0.61^**^	−0.12	0.59^**^	0.80^**^	0.50^**^	0.89^**^	0.62^**^	1
Trait anxiety	0.32^**^	0.41^**^	−0.32^**^	0.01	0.34^**^	0.37^**^	0.43^**^	0.46^**^	0.29^*^	0.22^*^

* *p < 0.05;*

** *p < 0.01. MAD_T1, mean absolute deviation for all trials in emotional cognitive task; MAD_T2, mean absolute deviation for all trials in non-emotional cognitive task; RTCV_T1, reaction time coefficient of variation in emotional cognitive task; RTCV_T2, reaction time coefficient of variation in non-emotional cognitive task; SD_T1, standard deviation of reaction times in emotional cognitive task; SD_T2, standard deviation of reaction times in non-emotional cognitive task; RT_T1, mean reaction time for all trials in emotional cognitive task; RT_T2, mean reaction time for all trials in non-emotional cognitive task; ACC_T1, accuracy in emotional cognitive task; ACC_T2, accuracy in non-emotional cognitive task*.

## Discussion

In the present study, patients with GAD demonstrated longer RT for both the emotional (presented in the emotional spatial-cuing task) and non-emotional conditions (presented in the non-emotional arrow flanker task) compared with non-clinical groups, indicating that GAD showed impaired processing efficiency indexed by RT (36). This is well-consistent with the previous findings that anxiety can impair top-down executive control to ignore task-irrelevant information (37, 38). In contrast, this phenomenon is not found in the HTA group from non-clinical populations. A recent study by Yu et al. (27) also found that there are significant differences in processing efficiency between the HTA and GAD groups. Therefore, it is necessary to further examine the existing opinion that features of executive control in subclinical samples can be extended to the corresponding clinical disorder (39).

For performance effectiveness (i.e., the quality of performance), patients with GAD exhibited lower correct rates than the other two groups in the emotional rather than the non-emotional task. This likely implied that the influence of trait anxiety on executive function is more pronounced under threat-related conditions. These results also support attentional control theory, suggesting that elevated anxiety may not impact cognitive functions in the absence of threat or substantial cognitive load (23, 26). It should be noted that the mean correct rates for all groups in arrow flanker task are more than 99%. Therefore, the inference about intact performance effectiveness for patients with GAD could only be applied to tasks with neutral stimuli and low cognitive load (40). Nevertheless, our findings also lent to strong support for the recent viewpoint that patients with GAD indeed exhibited obvious impaired executive control compared with non-clinical individuals (27).

In this study, analyses of variance revealed that patients with GAD and non-clinical persons could overall be effectively discriminated by MAD, RTCV, and SD in the emotional spatial-cuing task, and they could be effectively discriminated by RTCV and SD in the non-emotional arrow flanker task. Our results indicate that increased IIV in GAD is primarily related to inefficient prefrontal neural processing (41, 42). This is also consistent with the finding that higher levels of executive control result in lower levels of IIV (43). Discriminative power of MAD is unstable, which may lay in the fact that executive control functions of different anxious groups are more difficult to distinguish under the low cognitive load condition. The applicability of the current results needs further verification in difficult tasks requiring more cognitive resources.

Our study demonstrates, for the first time, a link between behavioral IIV and GAD. These data are in support of the viewpoint proposed by Bellgrove et al. that abnormally increased variability is an important index of disorders of executive/attentional control (44). The results based on patients with GAD in our study are also quite in accordance with the findings across studies that indicate IIV is associated with cognitive impairment in other psychiatric disorders (13, 14, 36). These findings may help to understand the associations of anxiety and attentional control. Furthermore, our results demonstrate that SD and RTCV have better across-task reliabilities than MAD. Partial correlation analyses among MAD, RTCV, SD, and mean RT suggest that compared to RTCV, SD and MAD are more easily affected by mean RT. Accordingly, RTCV is recommended as an excellent measure of IIV in this study. However, our results are inconsistent with the viewpoint by Saville et al. that SD or MAD is the best choice of parameter for measuring IIV (9). A possible explanation may lie in the fact that age, fluid intelligence, functional changes of frontal brain regions, and other factors have great impacts on cognitive performance (45). Ignoring the influences of these factors may lead to completely different conclusions. From the calculation method of the three parameters, MAD is most vulnerable to the influence of reaction time, followed by SD. Therefore, we still believe that RTCV is more reliable although it needs more evidence.

Nevertheless, our research replicates the findings from Lövdén et al. (17), which state that cognitive variability may serve as an early warning of imminent cognitive decline. Haynes and colleagues also assert that measures of RT variability have considerable potential in clinical contexts as they may aid identification and diagnosis of a range of neurobiological disorders (46). Our results provide new evidence for this view. Compared with the LTA and HTA groups, patients with GAD showed increased IIV in the emotional spatial-cuing task and the non-emotional arrow flanker task. These findings afford empirical evidence for existing accounts suggesting that the increased IIV of RT is a stable feature of clinical disorders observed across diverse tasks and methods (11). Developing new tools for assessing executive control is important given the clinical relevance of this psychological construct in the development and maintenance of psychopathology.

It is worth noting that there are some shortcomings in this study. First, difficult tasks requiring more cognitive resources should be adopted to further test whether trait-anxious individuals from non-clinical persons exhibit abnormal increase IIV. Second, only three parameters (MAD, RTCV, and SD) were compared in the present study. More parameters should be considered to optimize the measurement method of IIV. Third, considering that IIV is a relatively stable factor, only the impact of trait anxiety on participants' performance was examined in our study. Whether and how state anxiety affects participants' performance was not explored in our study. This is an interesting direction for future studies. Fourth, four participants of the GAD group completed less than high school education and none of the LTA and HTA groups. There might be sampling bias in this study. Finally, all the participants in the current study are Han nationality, and the small sample size may undermine the significance of group effects. Accordingly, a larger sampling is needed to replicate these results in other cultural or ethnic contexts in future studies.

Notwithstanding these limitations, several key implications can be drawn to better understand the characteristics of executive/attentional control of high anxious individuals. Due to the increased variability in patients with GAD in the present study, combined with previous studies on ADHD, PTSD, depressive disorder, etc., it indicates that increased variability appears a crucial marker of clinical disorders observed across diverse tasks and methods. IIV represented by RTCV is found to be objective, reliable, feasible to operate, and less influenced by RTs. Therefore, it is expected to serve as a supplement for existing clinical assessment to distinguish individuals with psychiatric diseases. Also, our study could shed light on that IIV might be an indicator of treatment effects for psychiatric disorders. In future research, it is necessary to develop the standard operating procedures for measurement of IIV with detailed guidelines and norms to interpret results in specific groups of people.

## Data Availability Statement

The raw data supporting the conclusions of this article will be made available by the authors, without undue reservation, to any qualified researcher.

## Ethics Statement

The studies involving human participants were reviewed and approved by the Ethics Committee of Army Medical University, Chongqing, China. The participants provided their written informed consent to participate in this study. The patients/participants provided their written informed consent to participate in this study.

## Author Contributions

YY and ML designed research. YY, HX, YX, and FL performed experiments and collected data. YY, HX, and YX analyzed data. YY, FL, and ML wrote and revised the paper. All authors contributed to the article and approved the submitted version.

## Funding

This research was supported by Social Science Planning Project of Chongqing (No. 2020YBSH107) and National Social Science Foundation of China (No. 19BSH132).

## Conflict of Interest

The authors declare that the research was conducted in the absence of any commercial or financial relationships that could be construed as a potential conflict of interest.

## Publisher's Note

All claims expressed in this article are solely those of the authors and do not necessarily represent those of their affiliated organizations, or those of the publisher, the editors and the reviewers. Any product that may be evaluated in this article, or claim that may be made by its manufacturer, is not guaranteed or endorsed by the publisher.
